# Results-Based Financing (RBF) Implication on the Availability of Essential Health Commodities in Primary Health Facilities: A Retrospective Pre- and Post-intervention Study in Tanzania

**DOI:** 10.7759/cureus.67271

**Published:** 2024-08-20

**Authors:** George M Ruhago, Aneth Wibroad

**Affiliations:** 1 School of Public Health and Social Sciences, Muhimbili University of Health and Allied Sciences, Dar es Salaam, TZA; 2 Phamaceutical Services, Ministry of Health, Dodoma, TZA

**Keywords:** health financing, medicine, supply chain management, essential health commodities, result based financing

## Abstract

Background: The health commodities supply chain is considered an important building block of any functional health system. There is a suboptimal availability of various health commodities in Low and Middle Income Countries (LMIC). Result Based Financing (RBF) is the innovation in health system financing that links financing with results. In Tanzania, a supply chain component was added to the RBF implementation in 2016 with the purpose of improving essential health commodities availability. This study aimed to evaluate the effects of RBF on essential health commodities in Tanzania, i.e., health commodities that have a high impact on the population's priority health care needs. They must be available all the time and be affordable in Tanzania.

Methods: The study employs a retrospective pre- and post-intervention design with a comparison group, ensuring a systematic approach to data collection and analysis. The intervention area was the Medical Store Department (MSD) Mwanza zone, while the comparison was the Moshi MSD zone. Data was extracted from the electronic Logistic Management Information System (eLMIS) on a quarterly basis at the health facilities from April 2016 to September 2018. A Repeated Measure Analysis of Variance (RMA) analysis was carried out.

Results: The study showed that the average availability of essential health commodities at the facilities in the RBF implementing zone was higher than in the non-implementing zone (50 vs 33). Similarly, there was a significant effect of RBF on the essential health commodities availability at health facilities F (9, 414) = 12.83, p = 0.0000.

Conclusion: Result Based Financing has a positive impact on the availability of essential health commodities in the implementing areas.

## Introduction

Health commodities, including medical products, vaccines, and technologies are one of the key World Health Organization (WHO) health system building blocks [1]. A well-functioning health system ensures equitable access to essential medical products, vaccines, and technologies of assured quality, safety, efficacy, and cost-effectiveness, with scientifically sound and cost-effective use [2].

Availability of essential health commodities remains a challenge in many low- and middle-income countries. The scoping review of essential health commodity availability in low- and middle-income countries using the WHO list of essential medicines indicated sub-optimal availability [3]. Another survey in six low- and middle-income countries found the availability of 32 medicines to treat chronic diseases was, on average, below 8% with percentages varied between countries. Stock-outs due to poor estimates of consumption, lack of local manufacturing, and cash-flow constraints were highlighted as possible reasons [4]. Unavailability of medicine is associated with poor patients ‘satisfaction and trust to the care [5]. A study at 17 health facilities in Tanzania indicated that the availability of oxytocin was 28%, while less than 50% of health facilities had intravenous (IV) fluids. Shortage of medicines adversely affected the provision of emergency obstetric care, demoralized health providers and decreased clients' trust in the health system [6].

Result based financing (RBF) is the innovation in health system financing which links financing with results and, hence, accelerates health system strengthening [7]. It involves “a cash payment or non-monetary transfer made to a national or sub-national government, manager, provider, payer, or consumer of health services after predefined results have been attained and verified [8]. The underlying assumption is that these financing schemes will motivate and enhance providers to pursue agreed targets aggressively and hence achieve quality performance targets. Studies show that it has been successful in changing healthcare workers’ behaviour and improving their performance [9].

This type of financing has been attracting global attention as a strategy to strengthen the healthcare system [10]. RBF has been introduced to several Sub-Saharan African countries aiming at increasing utilization, quantity, and quality of primary healthcare services by motivating healthcare workers by providing incentives upon agreed verified performance [11]. However, the current evidence on the effectiveness of this scheme in improving the performance of the health commodity supply chain and subsequently the availability of health commodities in different settings is limited. A Cochrane review of studies conducted in LMIC showed the desirable effect of RBF on medicine availability, although the certainty of evidence was low [12]. Studies in Tanzania and Congo have shown the positive impact of RBF on essential medicine availability [13,14]. In Burkina Faso and Afghanistan, no impact was reported [15,16].

Supply chain for health commodities

The Tanzania supply chain for health commodities is divided into three main levels. Firstly, health facilities are mandated to forecast their needs depending on the health services rendered to their clients. Secondly, regional and council level (managed by the Regional Health Management Teams (RHMTs) and Councils Health Management Teams (CHMTs), which oversee the supply chain issues in their respective regions and councils with the major role of supportive supervision. Thirdly, at the central level, Ministry of Health (MOH) and Medical Stores Department (MSD) have the major role of developing guidelines, protocols, resource mobilization, procurement, storage and distribution of health commodities [17]. The Medical Stores Department (MSD) is responsible for Procurement, Storage, and Distribution of health commodities in public health facilities. Currently, MSD comprises eight zones and two sales points. Health commodities flow from the central warehouse to the zonal and sales point warehouse then to the health facilities [18].

RBF in Tanzania

Based on the success stories of performance-based financing schemes in Rwanda and other countries, Tanzania piloted Pay for Performance (P4P) scheme in the Pwani region in 2011 with the aim of improving maternal and child health through motivating healthcare workers by providing incentives upon agreed performance indicators. The scheme showed a positive effect in improving performance by motivating workers [13].

Pay for performance was rolled out as Result Based Financing with support from the World Bank in 2015 in Shinyanga, Kagera, Pwani, Mwanza, Simiyu, Geita, Tabora and Kigoma regions. The criteria for implementing RBF in these regions were their poor health outcomes and high poverty index [17]. RBF is aimed at strengthening the health system by changing health workers' mindsets, increasing utilization of primary health services, and improving the quality and efficiency of health services [17]. To improve essential health commodities availability, which in turn strengthens the health system, the supply chain component was also included in Tanzania's RBF scheme in 2016 [17]. Central warehouse, transport unit, and vertical programs have been included in the RBF scheme at MSD headquarters and Mwanza, Tabora, and Dar es Salaam zones. These zones were selected to match the regions where the MoH was implementing the RBF scheme for health facilities and CHMT [17].

Since the introduction of RBF in the Tanzania health commodity supply chain, there has been limited evidence on its effects on essential health commodities availability. One existing study evaluated the impact of RBF on essential RMNCH commodities in one region [13]. This study aims to assess whether the introduction of RBF to the public health supply chain has improved the availability of essential health commodities at health facilities.

## Materials and methods

Study design and area 

A retrospective pre- and post-intervention study design with a comparison group was employed. The study was conducted in two MSD zones which are Mwanza and Moshi. Mwanza was selected as a representative of the RBF-implementing zone and Moshi as a representative of the non-RBF-implementing zone. The Mwanza MSD zone supports four regions (Mwanza, Shinyanga, Simiyu and Mara) with a total of 1065 facilities, while the Moshi MSD zone supports three regions (Kilimanjaro, Arusha, and Manyara) with a total of 755 facilities. 

Sampling procedure

Purposive sampling was employed to select the Mwanza zone based on its longer implementation period therefore rich in data. Moshi zone was randomly selected from the list of five non-RBF implementing zones. From the Mwanza and Moshi zones, Mwanza and Arusha regions were randomly selected using a lottery approach. Then in those two regions, the Ilemela and Arusha districts were also randomly selected using the same approach. In those two districts (Ilemela and Arusha), all public health facilities were included in the study, i.e., District Hospitals, Health Centres and Dispensaries.

Profile of MSD zones and facilities studied

**Figure 1:**
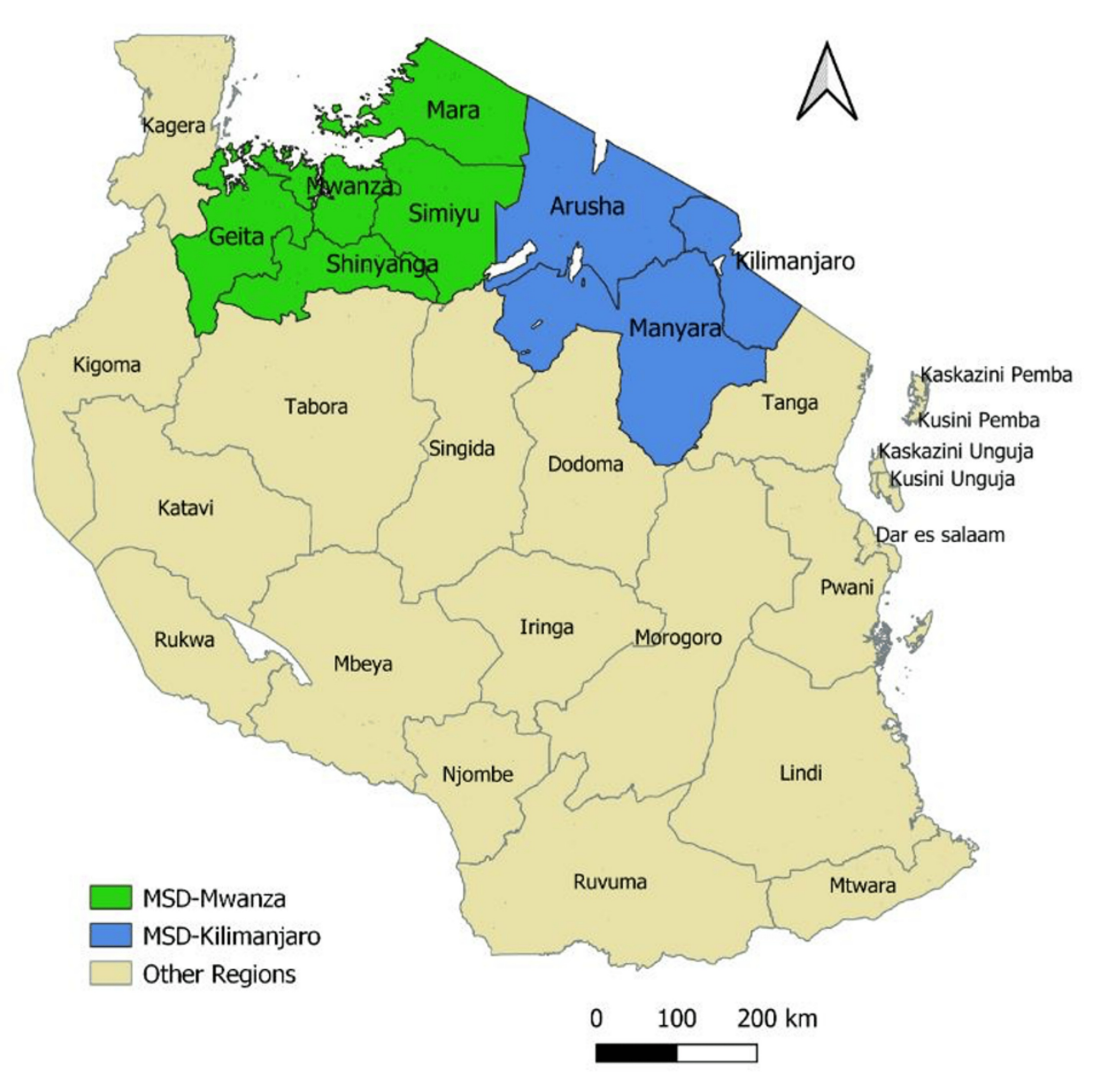
Regions supported by Mwanza and Moshi MSD zones MSD: Medical Store Department. The artwork is the authors' own creation.

MSD Mwanza zone was selected as RBF implementing zone and MSD Moshi as a non-RBF implementing zone. Mwanza zone supports Mwanza, Mara, Simiyu, Shinyanga and parts of Geita region. Moshi zone supports the Kilimanjaro, Arusha, and Manyara regions. Mwanza zone supports 1065 facilities while Moshi supports 755 facilities. Table 1 details the regions, councils, and public facilities supported by each zone. Figure 1 shows the regions in the map.

**Table 1:** Profile of Mwanza and Moshi MSD zones MSD: Medical Store Department.

Description	MSD Mwanza	MSD Moshi
Number of supported regions	4	3
Number of supported councils	32	17
Number of supported public health facilities	1065	755

Data collection

Data was extracted from the electronic Logistic Management Information System (eLMIS) for Ilemela and Arusha district health facilities. Data on availability of essential health commodities before and after RBF implementation in both Ilemela and Arusha district health facilities were extracted from 2016 to 2018. The eLMIS was used to obtain data on the availability of all selected ten essential health commodities (Table 2) below.

**Table 2:** Ten essential health commodities

SN	Health Commodities
1	Ferrous Sulphate + Folic Acid (FeFol)
2	Artemether (ALU),
3	Amoxycillin or Cotrimoxazole,
4	Albendazole or Mebendazole,
5	Oral Rehydration Salts (ORS)
6	Injection Ergometrine or Oxytocin or tabs Misoprostol
7	IV fluids (Dextrose 5% or Sodium Chloride + Dextrose)
8	Disposable syringes,
9	Malaria Rapid Diagnostic Test (MRDT)
10	Paracetamol

Ten essential health commodities (Table 2) prior defined by the Ministry of Health as a must-have list for all health facilities. The essential health commodities were regarded as available if the average month of stock was more than three months as indicated in Logistic System Standard Operating Manual 2018. The average availability of all ten selected essential health commodities was recorded from April to June 2016 before RBF implementation (pre-implementation). The post-implementation period was measured each quarter during RBF implementation from July 2016 to September 2018.

Data management and analysis 

All quantitative data were entered into 2016 Microsoft Excel and imported into STATA software version 15.1 (StataCorp LLC., College Station, TX) for analysis. A Repeated Measure ANOVA (RMA) analysis was carried out to examine the association between RBF implementation and the availability of essential drugs at health facilities [19]. The following assumptions were considered and met before and during each analysis (Table 3).

**Table 3:** Assumptions employed during repeated Measure ANOVA (RMA) analysis

SN	Assumptions
1	All dependent variables were measured at the continuous level (i.e., they are interval or ratio variables).
2	The independent variable is categorical, with three or more separate measurements of the same participants; performance was measured at ten different points.
3	No significant outliers in the related groups were encountered/observed
4	The distribution of the dependent variable in the two or more related groups was approximately normally distributed. This was checked using skewness and kurtosis tests
5	The variances of the differences between all combinations of related groups must be equal, known as sphericity

This was tested using Mauchly's test of sphericity. Mauchly's Test of Sphericity tests the null hypothesis that the variances of the differences are equal. Thus, if Mauchly's Test of Sphericity is statistically significant (p < 0.05), we can reject the null hypothesis and accept the alternative hypothesis that the variances of the differences are not equal (i.e., sphericity has been violated). When sphericity is violated, an adjustment factor called epsilon is applied to produce more valid F-values. These corrections/adjustments to combat the violation of the assumption of sphericity are the Greenhouse-Geisser correction and the Huynh-Feldt correction [20]. 

Ethical considerations

Ethical clearance was sought and obtained from Muhimbili University of Health and Allied Sciences (MUHAS) through the Institutional Review Board (IRB) no: DA.287/298/01A. Permission to collect data in health facilities was obtained from the regional and district authorities. The study did not collect any personally identifiable data as it was concerned with the availability of essential health commodities at the health facilities.

## Results

Distribution of health facilities in study areas

The majority of facilities in both zones were dispensaries (83%), and health centres accounted for the remaining 17% (Table 4). The proportion of facilities did not differ between RBF and non-RBF zones when the Chi-square test was done: X2 (1, N = 47) = 0.1384, p > 0.1384. At the time of the study, none of the districts had a hospital-level facility.

**Table 4:** Distribution of health facilities across districts involved in the study RBF: Result Based Financing

Type of facility	Non- RBF zone (Arusha DC) (N) (%)	RBF zone (Ilemela DC) N (%)	Total N (%)
Dispensary	27 (84%)	12 (80%)	39 (83%)
Health Center	5 (16%)	3 (20%)	8 (17%)
Total	32 (100%)	15 (100%)	47 (100%)

Association between RBF and availability of essential health commodities at the facilities

The study showed that the mean availability of essential health commodities in the RBF implementing zone is higher than in the non-implementing zone (50 vs 33) (Table 5). 

**Table 5:** Mean availability of essential health commodities for RBF and non-RBF implementing zones RBF: Result Based Financing

MSD Zone	Group	Commodities Availability (Baseline) (Mean)	Mean Commodities Availability (Post) (Mean)	(SD)	(N)
Mwanza	RBF	38.6	50.0	13.1	9
Moshi	Non-RBF	18.8	33.0	6.0	9

The values were measured every quarter for thirty months, and the initial values were the baseline. Therefore, repeated measure ANOVA (RMA) analysis was used to test the hypothesis that RBF improves the availability of essential health commodities at the facilities. 

Mauchly test of sphericity χ2(44) = 67.14, p=0.01 indicates a violation of sphericity (if p<0.05, sphericity is violated). Because sphericity is violated, we used the G-G correction factor in the interpretation. The results of the one-way repeated-measures ANOVA showed that there was a significant effect of RBF on commodities availability in facilities F (9, 414) = 12.83, p = 0.0000. Facilities in the RBF zone had a higher mean score on the availability of commodities compared with facilities not in RBF (Table 6).

**Table 6:** Availability of essential health commodities at the facilities (p<0.05)

	Sphericity Correction	Sum of Squares	df	Mean Square	F	P
Commodities availability measured in repeated times	None	27357.6	9	3030.7	12.83	0.0001
	Greenhouse-Geisser	27357.6	9	-	12.83	0.0001
	Huynh-Feldt	27357.6	9	-	12.83	0.0001
Residual	None	98081.35	414	236.91	(NA)	(NA)
	Greenhouse-Geisser	98081.35	414	12.83	(NA)	(NA)
	Huynh-Feldt	98081.35	414	12.83	(NA)	(NA)

## Discussion

This study has revealed that the availability of essential health commodities at health facilities in the RBF implementing zone is higher than in the non-implementing zone (50 vs 33). One-way repeated-measures ANOVA showed that there was a significant effect of RBF on commodities availability at health facilities. Our findings are similar to findings from a study by Binyaruka et al., which indicated that data from a before and after intervention of the pay for performance (P4P) scheme in Pwani region, Tanzania. The P4P was associated with an 8.4 percentage point increase in the availability of 37 medicines combined (P = 0.002) and an 8.3 percentage point increase in the availability of medical supplies, although this was borderline significant (P = 0.050) [13].

A before and after study that was conducted in South Kivu, Democratic Republic of Congo (DRC), comparing findings before and after RBF intervention corroborated our findings that the availability of drugs improved by 12% [14]. Similarly, a qualitative study conducted in Cameroon involving fifty-five health facilities participating in the scheme indicated that the performance-based financing (PBF) program improved the perceived availability of essential medicines in three regions in Cameroon. According to respondents, the PBF intervention had a positive impact on the availability of a variety of drugs. Respondents reported that its effects progressively minimized stock out [21]. The similarities between our findings and the previously reported findings indicate the importance of introducing payment based on results and its influence on the availability of health commodities.

In Burundi, a study involving RBF rollout, pooled percentage point change in drug availability was 4% when compared with comparison provinces, the result was not statistically significant (P=0.492) (21). The possible explanation could be that drug availability was not an incentivized outcome that might have contributed to making the results non-significant. Other indicators showed improvement; performance-based financing increased the share of women delivering their babies in an institution by 22 percentage points, and the share of women using modern family planning services by 5 percentage points [22].

Various modes in which RBF improves healthcare performance indicators have been suggested; these include the healthcare providers receiving a portion of the RBF as incentives, improved motivation for better performance, and increased autonomy and accountability, which have been highlighted as other factors [23]. Providing incentives to regional and district health teams through RBF has been shown to improve accountability due to increased supervision and collaboration [24]. Specific to drug availability, the autonomy to use bonus funds and other revenue to purchase essential health commodities has been cited as a potential pathway for reducing stockouts [13].

Contrasting findings have been documented. A cluster randomized controlled trial conducted in Afghanistan to determine if RBF could improve maternal and child health services found no effect on drug availability. The pharmaceutical and vaccine availability index between RBF and non-RBF facilities was not different (80.4% vs 79.8%, p=0.4) (18). Authors attributed this limited RBF impact to the implementation challenges as the study was rolled out in phases, delays in payment of bonuses and workers complained the amount of bonus was little/insignificant [15]. Similarly, in Burkina Faso, RBF had no impact on the availability of essential medicine. The study compared intervention and control districts and employed difference-in-difference regression analysis [16]. Lack of financial and management autonomy, rigid health commodity supply structure, payment delays and offering full RBF payment even if some indicators have not performed were cited as reasons for ineffective RBF performance [16].

While interpreting the results of this study, it should be noted this study encountered some limitations. The study faced two major challenges: poor documentation of supply chain data at both zones and facility levels. To mitigate this limitation, we opted to triangulate data from different sources such as District Health Information Software (DHIS2), LMIS books, and RBF verification reports. Further, the results of this study might have been influenced by other strategic initiatives that were undertaken by the Government during the same period to improve health commodity supply chain performance at MSD and subsequent availability of health commodities. Such initiatives include the introduction of innovative financing such as direct health facility financing, whereby funds are directly sent to the health facilities at all levels instead of all funds being spent at the council headquarters [1,2]. The introduction of a prime vendor system to provide a backup source of health commodities, in case MSD is out of stock [25].

## Conclusions

The study showed that when RBF was introduced and implementation influenced the availability of essential health commodities in the implementing districts compared with non-implementing districts. Based on the study findings, a scale-up of the RBF to other zones and regions to improve the availability of essential health commodities in health facilities is recommended. To ensure the success of the RBF rollout, a supportive/conducive environment for implementation should be facilitated; payment delays should be avoided, reimbursement structure based on performance should be clear, and facilities should have some autonomy on how to use RBF funds to further improve or sustain performance. Future research should aim at impact evaluation, employing randomized control studies to further enrich the benefits of the RBF to the health outcomes and health systems at large.
